# Microbial Degradation of Hydrocarbons—Basic Principles for Bioremediation: A Review

**DOI:** 10.3390/molecules25040856

**Published:** 2020-02-14

**Authors:** Łukasz Ławniczak, Marta Woźniak-Karczewska, Andreas P. Loibner, Hermann J. Heipieper, Łukasz Chrzanowski

**Affiliations:** 1 Institute of Chemical Technology and Engineering, Poznan University of Technology, Berdychowo 4, 60-965 Poznan, Poland; marta.w.wozniak@doctorate.put.poznan.pl (M.W.-K.);; 2 Department IFA-Tulln, Institute of Environmental Biotechnology, BOKU - University of Natural Resources and Life Sciences, Vienna, Konrad-Lorenz-Straße 20, 3430 Tulln, Austria; andreas.loibner@boku.ac.at; 3 Department of Environmental Biotechnology, Helmholtz Centre for Environmental Research—UFZ, Permoserstraße 15, 04318 Leipzig, Germany; hermann.heipieper@ufz.de

**Keywords:** bioaugmentation, biodegradation, biofilm, biosurfactants, biostimulation, crude oil, hydrocarbons, marine and terrestrial contamination, nutrient limitation, PAHs, surfactants

## Abstract

Crude oil-derived hydrocarbons constitute the largest group of environmental pollutants worldwide. The number of reports concerning their toxicity and emphasizing the ultimate need to remove them from marine and soil environments confirms the unceasing interest of scientists in this field. Among the various techniques used for clean-up actions, bioremediation seems to be the most acceptable and economically justified. Analysis of recent reports regarding unsuccessful bioremediation attempts indicates that there is a need to highlight the fundamental aspects of hydrocarbon microbiology in a clear and concise manner. Therefore, in this review, we would like to elucidate some crucial, but often overlooked, factors. First, the formation of crude oil and abundance of naturally occurring hydrocarbons is presented and compared with bacterial ability to not only survive but also to utilize such compounds as an attractive energy source. Then, the significance of nutrient limitation on biomass growth is underlined on the example of a specially designed experiment and discussed in context of bioremediation efficiency. Next, the formation of aerobic and anaerobic conditions, as well as the role of surfactants for maintaining appropriate C:N:P ratio during initial stages of biodegradation is explained. Finally, a summary of recent scientific reports focused on the removal of hydrocarbon contaminants using bioaugmentation, biostimulation and introduction of surfactants, as well as biosurfactants, is presented. This review was designed to be a comprehensive source of knowledge regarding the unique aspects of hydrocarbon microbiology that may be useful for planning future biodegradation experiments. In addition, it is a starting point for wider debate regarding the limitations and possible improvements of currently employed bioremediation strategies.

## 1. Introduction

To date, petroleum hydrocarbons are still among the major and most commonly occurring environmental pollutants [1]. Therefore, it is not surprising that the current view of hydrocarbons is affected by public concerns regarding contamination with crude oil-related products [2]. The production of crude oil, its transport, chemical processing and distribution are considered as the main sources of anthropogenic hydrocarbon pollution [3]. It is of common knowledge that hydrocarbons are toxic substances that exert a negative impact on the environment [4]. The fact that they may be utilized as a substrate by living organisms is usually considered as a unique trait, and isolation of hydrocarbon degraders is often treated as an extraordinary finding. Since microorganisms possess the ability to decompose hydrocarbons as an energy source; their application in bioremediation processes is a natural consequence. Nevertheless, although biodegradation has been recognized as a feasible method to remediate the polluted environment and a vast amount of research carried out in this field considerably improved our understanding of this process—there is still a need for further research. Furthermore, despite the fact that the mechanisms of hydrocarbon biodegradation processes are known, there are still numerous misconceptions regarding the relation between microorganisms and hydrocarbons, which result in the lack of a uniform theory. In order to fully comprehend the depth of the interactions between microorganisms and hydrocarbons, it should be remembered that the history of petroleum transgresses the issues of the modern world. As such, these interactions are not limited to accidental oil spills [5].

Since when do hydrocarbons exist, and when did microorganisms come in contact with them? Did microorganisms have to adapt to the utilization of hydrocarbons present in crude oil, or did they already possess this trait? Is the ability to biodegrade hydrocarbons rare, or is it prevalent in microbial populations? How do microorganisms function in a hydrocarbon-rich environment? What are the most important limitations for hydrocarbon biodegradation processes? Providing answers to the above-mentioned questions will allow to establish the main short-comings of currently employed decontamination methods based on biodegradation and identify crucial areas for future considerations.

The aim of this mini-review is to provide a factual background regarding the involvement of microorganisms in the formation of crude oil and its subsequent biodegradation. Additionally, this review outlines the factors which limit the growth of bacteria in hydrocarbon-rich environments. Finally, the strategies used for enhancement of hydrocarbon decontamination processes have been evaluated based on recently published reports.

## 2. The Inseparable Bond between Hydrocarbons and Microbes—When, Where and How?

According to the current assumptions, the age of the Earth is estimated at 4.5 billion years [6]. For the majority of this time, the inhabitants of Earth existed in the form of simple, unicellular organisms. A fundamental change occurred during the Cambrian period (approx. 545 million years ago), which is often referred to as the ‘Cambrian explosion’ due to the magnitude of occurring changes [7]. During the subsequent 20−25 million years, complex and multicellular organisms started to emerge and appear on a mass scale. While this phenomenon is significant in terms of several aspects, it also resulted in the inevitable increase of biomass generation and formation of its deposits in sediments. This simple fact is the starting point—it is from this moment that the conditions for the formation of hydrocarbons are met.

**Figure 1:**
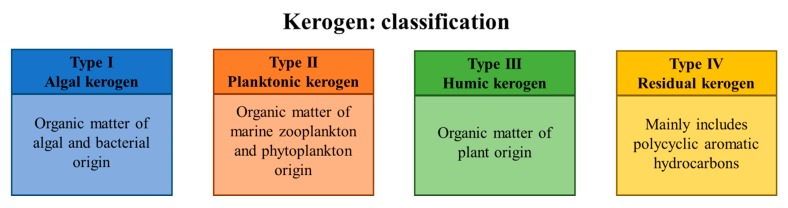
Classification of kerogen types based on their origin.

The currently accepted scenario regarding the origin of petroleum hydrocarbons is based on the following concept: fossil organic matter became entrapped in the source rock (kerogen) and underwent several stages of transformations [8]. From a chemical point of view, the substrates are a mixture of high molecular weight organic compounds formed due to the degradation of natural polymeric substances present in residual biomass. Depending on its origin and the potential to form petroleum hydrocarbons, kerogen is classified into four types (Figure 1). Thermal maturation of kerogen involves: (i) diagenesis—a relatively brief period of biological degradation, (ii) catagenesis—geothermal degradation and cracking and (iii) metagenesis—further decomposition that mainly results in the formation of methane. Accumulation of gaseous products leads to the migration of maturated crude oil hydrocarbons from the source rock into the reservoir rock. This process is limited by the impermeable layer of rock (cap rock), and the resulting accumulation of hydrocarbons enables the formation of the oil reservoirs. Due to high porosity (0–40%, depending on rock type with a typical pore size of approx. 100 µm), the reservoir rock is characterized by a notable capacity to store liquids [9]. While the saturation of the reservoir rock with liquid hydrocarbons depends on their initial concentration, it cannot reach 100% due to the presence of residual water in the pores. Ultimately, the hydrocarbon/water interface is formed, and this allows for microbial growth [10].

Probing of crude oil in order to establish its age revealed that 60% of commercially important sources appeared approx. 180–80 million years ago (during the Jurassic and Cretaceous periods), while the oldest reservoirs were most likely formed in the Ordovician period (485 million years ago). From a microbial perspective, this translates to whole millennia which could be spent on adaptations to utilize hydrocarbons as energy sources and efficiently colonize such substrate-rich niches. The extent of biodegradation of currently exploited oil reservoirs has a direct impact on the quality of crude oil. As the petroleum compounds are utilized by different bacteria as a source of carbon and energy, a progressive depletion of light hydrocarbons (C1–C6) occurs, followed by the dissipation of saturated hydrocarbons and aromatics (C6–C15) [11]. As a consequence, the composition of oil is changed, and its viscosity is increased due to enrichment in heavy petroleum fractions. This, in turn, may notably limit the efficiency of the mining process. In extreme cases, the extensive biodegradation of oil resources renders the extraction process economically unjustified (with the exception of shallow bituminous sands). The standard protocol used for evaluating the quality of crude oil is based on the American Petroleum Institute (API) gravity parameter, which is an estimation of hydrocarbon density relative to water. This parameter is influenced by the concentration of specific hydrocarbons and corresponds to the degradation extent of crude oil. Since the microbial activity is limited by the geothermal, the reservoirs at a depth up to 3–3.5 km (temperature < 80 °C) are usually substantially enriched in heavy fractions, whereas oil resources rich in low molecular weight fractions are usually found at a depth which exceeds 3.5 km (temperature > 80 °C) [12]. Estimations of API gravity parameters during oil mining indicate that almost 50% of global oil resources should be classified as “heavy” or “extra heavy”. This clearly confirms that biodegradation of crude oil occurs even in pristine reservoirs and elucidates the interaction between hydrocarbons and microorganisms, as the latter begin to proliferate in the reservoirs as soon as the environmental conditions allow it.

However, crude oil does not necessarily remain in the reservoirs. Disruption of cap rock integrity (e.g., due to tectonic activity) may result in the leakage of oil [13], which can often be observed in the form of rock oils or hydrocarbon lakes. It is estimated that 600 kt of petroleum compounds are introduced into the environment per annum as a result of such natural discharges. It should be emphasized that this amount is roughly equal to the overall amount of oil contamination resulting from anthropogenic activity [11]. Usually, upon release from their entrapment, the hydrocarbons penetrate upwards. The most common scenario is the leakage of crude oil into aqueous systems, especially marine environments. Nevertheless, such spills are rarely noticed by the public opinion, due to the high activity of microorganisms that carry out the biodegradation processes. In cases of terrestrial systems, the migrating oil may reach even relatively shallow depths, impregnate the exposed rocks and form bituminous sands (which are present, e.g., in Alberta in Canada or the Orinoco bituminous belt in Venezuela) or asphalt lakes, such as the Pitch Lake, Trinidad and Tobago, the world’s largest asphalt lake [14]. Such accumulations are also susceptible to biodegradation, which further increases the viscosity of bituminous sands.

It is also imperative to remember that crude oil does not constitute the sole source of hydrocarbons in the environment. Several species of plants possess the ability to synthesize and excrete hydrocarbons in order to form protective layers of waxes (which, e.g., prevent water loss). These compounds are characterized by considerable chain lengths (from C15 to C38) and vast structural diversity. At the global scale, the amount of hydrocarbons produced by plants is by far higher compared to natural crude oil spills. For example, in the sole case of isoprene and monoterpene, their production reaches 600–800 Mt per annum [11]. Animal-derived hydrocarbons, such as insect waxes (mainly C21–C33 alkanes), extend the spectrum even further. A curious case involves microbial taxa, which are also capable of producing hydrocarbons. This mainly applies to methane and C2–C4 gaseous hydrocarbons, although more complex structures (such as acetylenes, isoprenoids, acyclic C10–C30 and cyclic hydrocarbons) may also be formed, depending on the type of microorganism and the environment. This issue was elucidated in a detailed summary by Wackett in 2010 [15]. Hydrocarbons produced by living organisms are ubiquitous, although generally present at low concentrations in the environment (ranging from ng/L to µg/L) [11].

In summary of data presented in this chapter, microorganisms play a major role during the initial stages of hydrocarbon formation (diagenesis) and significantly influence the ultimate composition of commercially available crude oil, as evidenced by its different classes. Moreover, since nonpetroleum hydrocarbons are ubiquitous in the environment at a low level, microbes may interact with them. Based on the above-mentioned examples, it can be concluded that the connection between petroleum and microorganisms is inseparable.

## 3. To Biodegrade or not to Biodegrade?

After establishing that microorganisms had sufficient time and opportunities to adapt to the utilization of hydrocarbons as an energy source, it is necessary to consider some additional aspects: Is this strategy actually worth the effort? Is the ability to biodegrade hydrocarbons a common feature or a unique privilege? What are the major limitations of this process?

The fact that hydrocarbons are so eagerly utilized by microorganisms as a source of energy can be explained by a simple comparison of energy values. Chemicals with highly reduced carbon backbones, such as hydrogen-rich alkanes and aromatic hydrocarbons, are thus potentially good electron donors. The net energetic gain (estimated as heat energy released during combustion) from the digestion of fats (lipids), proteins and carbohydrates amounts to 37, 17 and 17 kJ/g, respectively [16]. This explains why lipids are commonly used for energy storage in the majority of organisms. In comparison, the combustion of crude oil provides 42–47 kJ/g of energy, which clearly exceeds even that of fats [17]. Oxidation of aliphatic hydrocarbons, which is typically the initial stage of their biodegradation process, results in the formation of fatty alcohols, which are further oxidized into fatty acids—the latter being natural components of lipids. This stage requires some energy input, and the final gain will depend on the available electron acceptors, as well as the type of hydrocarbons subjected to breakdown. In cases of linear alkanes, it can be expected that this value will be considerably higher in comparison to branched alkanes, not to mention cycloalkanes. The situation will also be notably different in case of benzene, substituted benzene derivatives or polycyclic aromatic hydrocarbons. From this perspective, it is clear that straight-chain hydrocarbons present in the environment are perceived as an exceptionally attractive source of energy by any organism capable of their degradation; however, all types of hydrocarbons will be beneficial in terms of total energy gain.

As for the prevalence of the ability to degrade hydrocarbons, studies focused on catabolic activity of various microbial populations provide an interesting answer [18,19]. It was established that hydrocarbon-degraders are always present in a given population, regardless of whether its habitat was exposed to anthropogenic contaminations or not. This general rule in microbial ecology: “Everything is everywhere, but the environment selects” was already stated in 1934 [20]. Although microorganisms which possess genes associated with hydrocarbon degradation are widespread, their relative abundance is rather low (below 1% of the total population). This corresponds well with the previous statements regarding the facts that: (i) hydrocarbons may occur in the environment as a result of natural discharges or biosynthesis by various organisms and (ii) the concentration of “natural” hydrocarbons is low. Furthermore, the limited number of hydrocarbon-degrading microorganisms reflects the fact that other energy sources are more prevalent in the environment. However, especially for the main crude oil ingredients, aliphatic and monoaromatic hydrocarbons [21,22], well-regulated and fine-tuned catabolic pathways are already present, reflecting their long-existing occurrence in the evolution when compared to “new” developed catabolic genes and operons, respectively, encoding for only quite recently applied chemicals such as pesticides (e.g., atrazine) or chlorinated hydrocarbons (e.g., highly chlorinated phenols and biphenyls) [23,24].

There are, however, exceptions to the statement given above—bacterial blooms following oil spills. In cases when the concentration of hydrocarbons drastically increases, e.g., as a result of unintended oil spills (or in close proximity of natural seepages), the abundance of hydrocarbon-degrading bacteria grows exponentially within a few days [25]. The described phenomenon was clearly visible in cases of major marine oil spills (e.g., the Exxon Valdez or Deepwater Horizon incidents in 1989 and 2010, respectively). Under such conditions, the carbon source is never a limiting factor. However, the rapid growth of hydrocarbon degraders is accompanied by the decrease of essential nutrients (i.e., nitrogen, phosphorous and iron), which results in the decline of the microbial bloom. This marks the major limitation of the hydrocarbon biodegradation processes.

**Figure 2:**
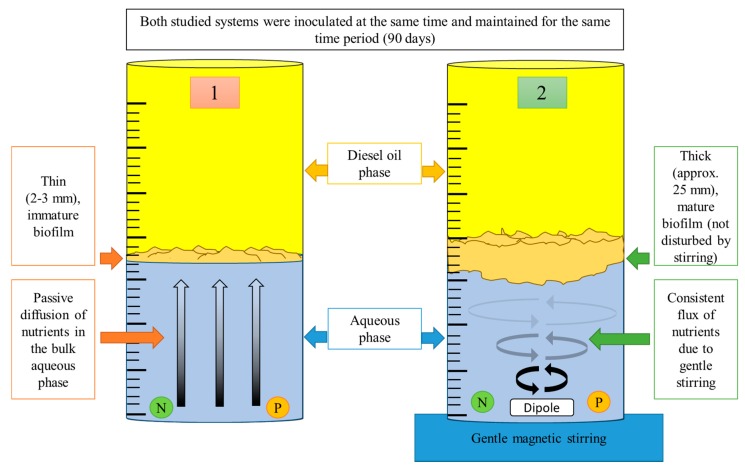
Experimental set-up of the experiment used to illustrate the influence of nutrient limitation on the development of bacterial biofilms at the diesel oil/water interface.

A simple experiment under laboratory conditions was carried out in order to verify the significance of nutrient limitations (Figure 2): bacterial biofilm growth was investigated at the oil-water interface in two 2-L glass cylinders (600 mL of mineral medium and 1400 mL of diesel oil). In one of the cylinders, the mineral medium was gently stirred using a magnetic stirrer (with no effect on the interface), which enabled the circulation of nutrients. The second cylinder was not equipped with a stirrer, and nutrient transport occurred solely due to passive diffusion. Both cylinders were inoculated with hydrocarbon-degrading communities (approx. 1 mL of cell suspension, cell density at 1 × 10^6^ CFU/mL) isolated from Gorlice (a location associated with the production of crude oil in Poland) at the same time [26].

**Figure 3:**
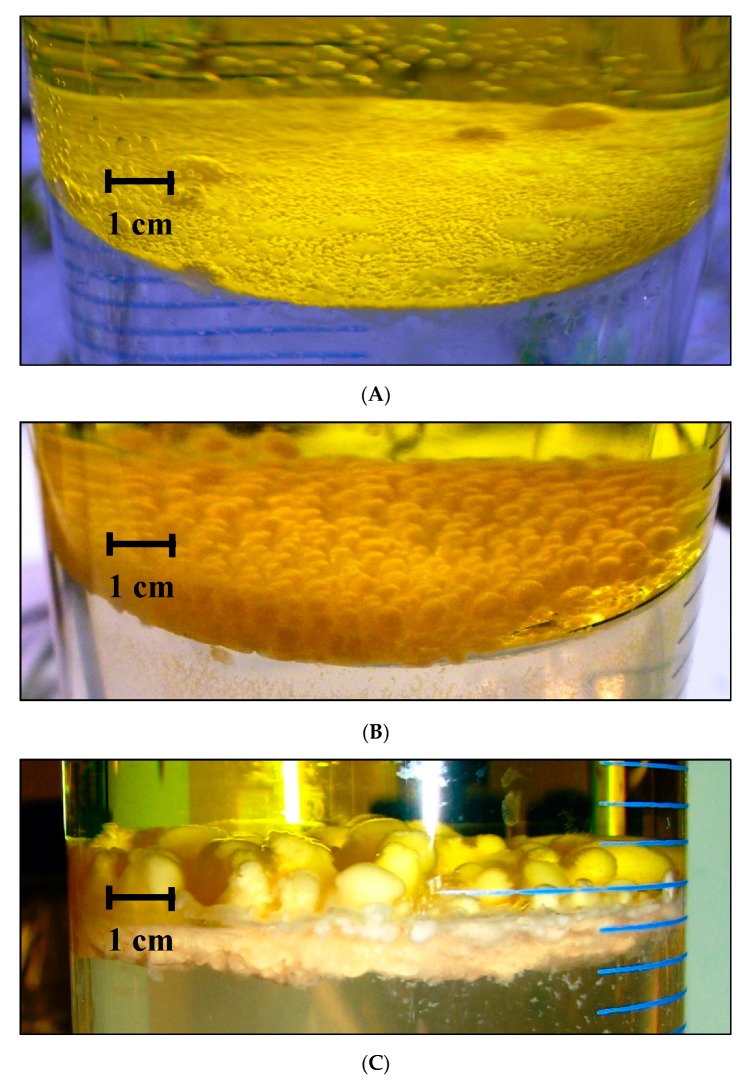
Biofilm formation at oil/water interface (diameter of the cylinder = 90 mm): (**A**)—Biofilm growth under diffusion-limited conditions (2–3 mm, 60 days after incubation). (**B**)—Biofilm growth under stirring, without limitations caused by diffusion (20 mm, 60 days after incubation). (**C**)—Development of mature biofilm structure without limitations caused by diffusion (25 mm, 90 days after incubation).

The differences in growth rates were clearly visible after two weeks following incubation (Figure 3). After approx. two months, the bacterial biofilm present in the cylinder without stirring achieved a thickness of 2–3 mm with small pseudo-mushroom structures. In contrast, the biofilm in the cylinder with stirring was characterized by a thickness of 20 mm and notably larger pseudo-mushroom structures, which fully maturated after 90 days.

**Figure 4:**
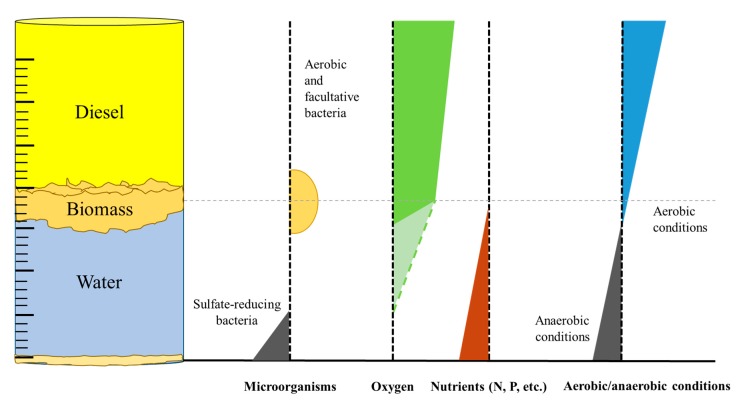
Profiles which represent microbial growth and the concentration of oxygen and nutrients (nitrogen, phosphorous, etc.), as well as the changes of redox potential in an oil/water system. (The dashed line in the oxygen profile represents reduced depletion in cases of immature biofilms and/or limited microbial growth.)

This experiment is a very good representation of biological processes which occur in fuel tanks. Contrary to common beliefs, the solubility of oxygen in most hydrocarbon mixtures is higher by even one order of magnitude compared to water (e.g., 15.7 mM in hexane and 8.7 mM in toluene compared to 1.3 mM in water [11]). Hence, the biofilm growing on the oil-water interface in oil tanks consists mostly of aerobic and facultative aerobic species that deplete the oxygen and nutrients, which may result in a progressive alteration of the bacterial community structure [27,28,29]. This results in strictly anaerobic conditions at the bottom of the cylinders. In consequence, the bottom of the aqueous phase is a perfect environment for sulphate-reducing bacteria, which are commonly associated with the microbial corrosion of carbon steel due to the generation of H_2_S (as presented in Figure 4).

Proliferation of bacteria at the oil-water interface is well-known in numerous sectors of the industry as a source of several operational issues—the petroleum industry being no exception. The expansive growth of bacteria results in generation of biomass, which mechanically clogs the pipeline systems, filters, valves, etc. [30]. Long-term presence of biofilms might contribute to microbial corrosion of carbon steel leading to leakage of crude oil, fuels and processing waters. Finally, microbial contamination will be responsible for reservoir souring and plugging, resulting in poor recovery of oil. The presence of water exposes crude oil, crude oil-derived fuels and each element of the processing installation to negative effects of microbial activity. As a result, the use of microbial control has become a necessity for the petroleum industry. This can be achieved by using modified materials (e.g., coated pipes to inhibit bacterial adhesion, which works only as a short-term solution); physical methods (e.g., ultrafiltration and UV sterilization, which are not feasible on a mass scale) or chemical methods (addition of biocides). This corresponds to a single significant fact—crude oil (or any fuel for that matter) cannot be stored or transported without the use of bactericides, which further confirms that biodegradation of hydrocarbons is an integral part of microbial life [31].

## 4. Practical Approaches to Bioremediation of Hydrocarbon Contaminations—The Good, the Bad and the Still Developing

In accordance with the currently accepted environmental protocols, each spill of hydrocarbons causing a concentrated contamination should be swiftly removed in order to minimize the negative effects on higher organisms, including humans. In cases where the on-site conditions are appropriate for microbial growth, natural processes of hydrocarbon biodegradation are initiated—this phenomenon is often referred to as natural attenuation (or, more specifically, intrinsic biodegradation) [32]. However, the limited kinetics of such processes often correspond to their considerable duration. Hence, although the microbial ability to biodegrade hydrocarbons is ubiquitous, improvement of its rate is crucial in order to efficiently carry out biological remediation [33]. The most commonly employed means of enhancing the biodegradation efficiency have been discussed below based on review of the recent literature reports (years 2017–2019) which have been listed in Table 1.

**Figure 5:**
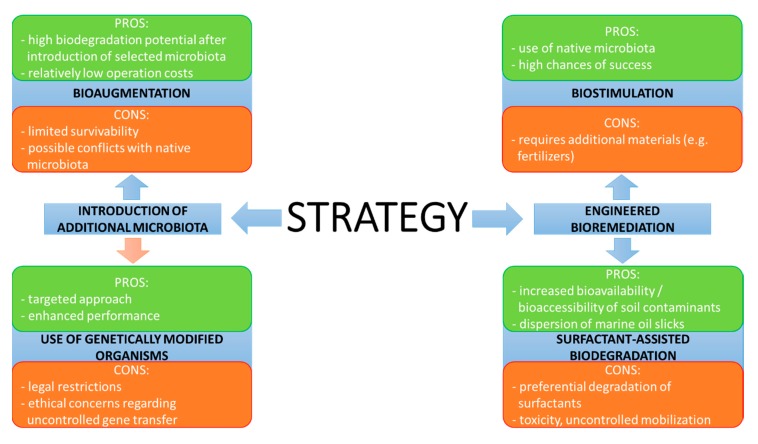
Overview of the most commonly applied strategies used to enhance hydrocarbon biodegradation processes for environmental clean-up.

The most popular strategies used to improve the efficiency of hydrocarbon dissipation include the introduction of additional microorganisms into the contaminated site or engineered bioremediation, which corresponds to processes focused on the intensification of biodegradation efficiency by the introduction of additives and ensuring optimal conditions for microbial growth (Figure 5).

Introduction of additional microbiota may be realized using two approaches: bioaugmentation and the use of genetically modified microorganisms. Currently, the latter solution, which relies on the introduction of genes relevant for hydrocarbon biodegradation pathways, is of limited practical value, mainly due to restricted legislation. For example, the introduction of genetically modified organisms in EU countries is subjected to strict regulation based on Directive 2001/18/EC of the EU Parliament. Additionally, public concerns, as well as issues with control (e.g., horizontal gene transfer phenomena) and maintaining genetic stability, further limit the applicability of this approach. Hence, this option is currently treated as a scientific curiosity and not an actual alternative that may be applied worldwide (as indicated by the red arrow in Figure 5). This is evidenced by the lack of studies regarding the application of genetically modified hydrocarbon degraders for actual bioremediation in Table 1. Bioaugmentation is a concept that has attracted much attention. It is based on the introduction of selected microbial species specialised in the biodegradation of specific compounds directly into the contaminated site. While the idea is potentially promising, the investigations focused on the practical application of bioaugmentation revealed several flaws [58]. Bioaugmentation seems only to be applicable in cases of very specific pollution and/or environmental conditions and when pollutants are present in very high concentrations [59]. The main issue is associated with the fact that the selected microorganisms often fail to proliferate in the area of their introduction. This may result from the fact that screening procedures are usually carried out under laboratory conditions, which do not reflect the actual environmental factors at a given site, or from antagonistic interactions with autochthonous populations. Another downside is the possible decrease of biodiversity as a result of the introduction of external species [42]. In this regard, a strategy named autochthonous bioaugmentation seems to be an option with a higher rate of success. The idea is based on the isolation of potent degraders from native microbial populations [60], their subsequent cultivation under laboratory conditions and re-introduction into the area of their origin. Recent reports confirm the feasibility of this approach [34,36]. The impact of autochthonous bioaugmentation can be further improved by the selection of most potent degraders based on metagenomics profiling [52], although it should also be mentioned that improvement of hydrocarbon removal efficiency may also occur after the introduction of strains which do not directly participate in the biodegradation process [35]. Despite the fact that autochthonous bioaugmentation may be more feasible, this approach is also often employed without proper identification of species present in the microbial population. As such, this solution may raise concerns, since several active hydrocarbon-degraders belong to pathogenic or opportunistic species and re-introduction of increased biomass that includes such microbiota would be an issue in terms of biosafety. The effect of bioaugmentation can also depend on the duration of the process—the strategy may result in enhancement during the initial phases of biodegradation, whereas it seems to be less effective during the latter stages of long-term treatment processes [38] or even result in inhibited soil respiration [37].

One of the most basic engineered bioremediation techniques, namely biostimulation, relies on the application of nutrients, terminal electron acceptors and additives, which stimulate the activity of native degrading microorganisms, as they are present in every site. The increased concentration of microbial cells results in a direct increase of the biodegradation rate. This approach directly corresponds with the previously mentioned limitation caused by an unbalanced C:N:P ratio. Even a minor lack of P results in a considerable amount of residual hydrocarbons, which on a mass scale is visible as a low biodegradation rate. This can be resolved by the addition of N and P sources, e.g., common fertilizers [44]. Currently, biostimulation is often combined with the previously mentioned bioaugmentation as a joint strategy to ensure high biodegradation efficiency [54,55,56]. In most cases, this approach was successful, although there are also reports regarding failed attempts [53]. In addition, several studies indicate that in the framework of such combined approaches, biostimulation is the main driving force [54,55]. The comparison of both strategies based on the reports listed in Table 1 indicates that biostimulation allows to achieve superior results, whereas the contribution of bioaugmentation was lower [40,42]. An interesting idea is to introduce the species used for bioaugmentation in an immobilized form, using natural carriers that also serve as sources of nutrients [41]. However, it should be emphasized that the type of amendment should be carefully selected, as some forms of nutrients may stimulate the process while others result in decreased performance [43].

It should be emphasized that the efficiency of biodegradation processes significantly differs in cases of aqueous and terrestrial environments. The treatment rate is notably lower in cases of the latter, due to the complexity of the soil matrix causing a lower bioavailability of the compounds. Typically, the soil should be viewed as a system consisting of mineral and organic components. The mineral components may vary in terms of porosity, which affects both the bioavailability and bioaccessibility of hydrocarbons via sorption/desorption [61]. Organic components (mainly humic acids) serve as a “sponge” or a “shuttle”, which controls the concentration of hydrocarbons and lowers their toxicity [62]. The third factor of great importance is the changing water content, which may influence the availability of water to microorganisms as well as the concentration of nutrients, dissolved oxygen and contaminants [11].

**Figure 6:**
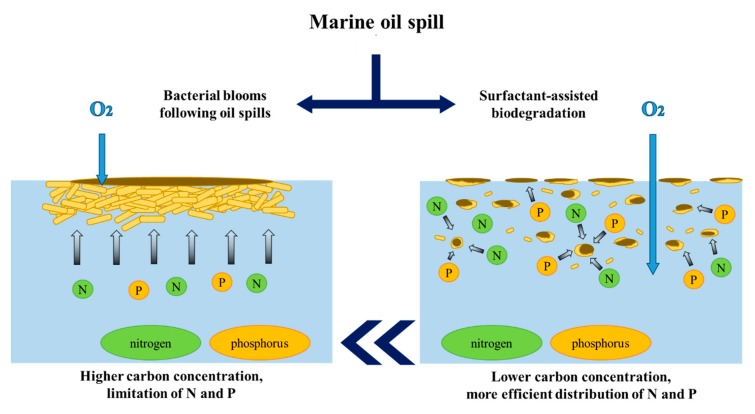
Distribution of nutrients (nitrogen, phosphorous, etc.) during marine oil spills in cases of bacterial blooms (**left**) and after applications of surfactants (**right**).

Surfactant-assisted biodegradation is a different variant of engineered bioremediation that may be employed in order to overcome the above-mentioned limitations in terrestrial systems. The basic idea behind the introduction of surfactants into hydrocarbon-contaminated soil is to enhance the bioavailability and bioaccessibility of such pollutants to microorganisms [63]. This approach is also used in cases of marine environments to disperse the oil slick into fine droplets, increasing the contact area between oil and water and allowing for an improved relative C:N:P ratio (as presented in Figure 6). Due to the dispersion of the oil phase into fine particles by surfactant molecules, the nutrients are utilized in a more efficient manner at the same concentration (the nutrient-driven limitation of bacterial growth is notably reduced).

Recently, this trend has evolved, and biosurfactants are employed more commonly as biodegradable and environmentally friendly alternatives to synthetic surfactants [50] (as can be observed based on Table 1). This concept has gained increasing popularity, and numerous studies employ externally added biosurfactants or biosurfactant-producing bacteria to improve hydrocarbon biodegradation processes [47]. Nevertheless, it should be noted that supplementation of surfactants and/or biosurfactants has often proven to be unsuccessful [45,46,48,57,64]. Among the possible causes indicated in recent reports, surfactants/biosurfactants introduced into the system were often treated as preferential sources of carbon and energy by the microorganisms compared to hydrocarbons [39,51]. This is especially plausible in cases of aged contaminations, which are rich in recalcitrant forms of hydrocarbons. Furthermore, formation of surfactant/biosurfactant micelles which surround hydrocarbons may facilitate their transport into the aqueous phase; however, it does not necessarily result in their immediate discharge into the bulk phase [65]. In consequence, the entrapment of hydrocarbons in micelles may ultimately hinder their bioavailability to microorganisms and result in decreased biodegradation rates [46,48]. The use of surfactants is also associated with other potential hazards [66]. Surface active compounds are often toxic to microbes (mainly in cases of cationic surfactants). Even biosurfactants, which are considered as nontoxic, may exhibit bacteriostatic or phytotoxic properties at high concentrations [67]. In addition, the application of surfactants/biosurfactants may result in the mobilization of hydrocarbons and their unintended transport to surrounding areas if appropriate protection measures are not employed. Furthermore, the interaction of surfactants with heavy metal ions may increase their mobility in soil and result in increased toxicity. The final effect of this approach seems to depend on the concentration of the surfactants [64] or biosurfactants [57], the concentration of hydrocarbons [44] and duration of the biodegradation process [49].

## 5. Summary

Clearly, there is a link between hydrocarbons and microorganisms that have evolved during millions of years of interactions. In consequence, the ability to degrade hydrocarbons is widespread among microbial populations.

The biodegradation processes will initiate intrinsically whenever the environmental conditions allow it. As such, biostimulation seems to be the safest strategy to improve hydrocarbon removal. Assuming that the compatibility of the introduced stimulant with the native microorganisms is tested and that reasonable doses of nutrients are introduced in order to maintain an optimal C:N:P ratio, there is little risk of failure of bioremediation processes involved.

Currently, bioaugmentation still requires further research in order to improve the odds of its successful application. Autochthonous bioaugmentation seems to be a more feasible variant. Future studies should focus on establishing protocols for the selection of the most appropriate strains based on the environmental conditions at the contaminated site and providing guidelines for the introduction of non-native strains. In this regard, the use of immobilized pollutant degraders seems potentially promising [68].

In order to properly employ (bio)surfactant-assisted biodegradation, there are numerous factors which should be taken into consideration, especially in soil systems. Based on the reviewed reports, it seems that administration of lower doses of (bio)surfactants in cases of high TPH content at initial stages of the biodegradation process results in improved efficiency. In this case, additional testing regarding toxicity, mobility of contaminants and preferential degradation of surfactants should also be carried out prior to any treatment processes.

Overall, it seems that despite the fact that hydrocarbons have been studied as environmental contaminants on numerous occasions and for several decades, there is still much to research.

## Figures and Tables

**Table 1:** Overview of recent studies regarding hydrocarbon biodegradation strategies.

Bioremediation Strategy	Contaminants	Test System	Removal Efficiency	Process Duration	Conclusions/Comments	Reference
Autochthonous bioaugmentation	Alkanes(initial concentration not specified)	Drill cuttings microcosms	35–66%	12 weeks	Consortia were isolated from drill cuttings, enriched and reintroduced. The consortia exhibited a high biodegradation potential towards several hydrocarbon substrates and the ability to produce biosurfactants. Enrichment of *Firmicutes* was observed.	Guerra et al. 2018 [34]
Autochthonous bioaugmentation	Phenanthrene (10 mg/L)	Bottle tests	>90%	6 days	Autochthonous bioaugmentation allowed to improve the biodegradation efficiency. The re-introduced autochthonous isolate did not directly participate in the biodegradation process, and the improvement was attributed to altered diversity of PAH degraders.	Li et al. 2018 [35]
Autochthonous bioaugmentation	Crude oil (10–50 g/kg)	Composting	60–91%	12 weeks	Re-introduction of two autochthonous isolates into the population allowed for successful bioaugmentation and improvement of the biodegradation process.	Koolivand et al. 2019 [36]
Bioaugmentation	Crude oil (TPH at 12 g/kg)	Soil microcosms	30–38%	182 days	The bioaugmentation initially improved the biodegradation efficiency; however, after 91 days, a significant decrease of soil respiration was observed with changes of the bacterial community composition.	Pacwa-Płociniczak et al. 2019 [37]
Bioaugmentation	Diesel oil and diesel/biodiesel blends (1% *v*/*w*)	Soil microcosms	88–97%	64.5 weeks	Bioaugmentation initially improved the biodegradation kinetics; however, there was no significant effect in the long term. Furthermore, the ratio of aliphatic to aromatic fractions remained unchanged regardless of the treatment used.	Woźniak-Karczewska et al. 2019 [38]
Bioaugmentation + biosurfactant/surfactant-assisted biodegradation	Pyrene (10 mg/kg)	Soil microcosms	60%	10 days	Bioaugmentation was successful. High biodegradation efficiency was observed in the case of unamended and surfactant (Brij-35)-amended soil samples. Supplementation with rhamnolipids inhibited the biodegradation process due to their utilization as a preferential carbon source.	Wolf and Gan 2018 [39]
Bioaugmentation + biostimulation	PAHs (1.5 g/kg)	Soil mesocosms	99%	56 days	Biostimulation successfully improved the biodegradation efficiency, whereas bioaugmentation did not significantly contribute to the process. Enrichment of the community in PAH-degrading species was observed.	Haleyur et al. 2019 [40]
Bioaugmentation + biostimulation	Crude oil (TPH at 20 g/kg)	Soil microcosms	36–51%	30 days	The highest biodegradation efficiency was achieved when bioaugmentation was carried out using an immobilized bacterial consortium, with *Eichhornia crassipes* dried straw acting as both a carried and additional source of C and N.	Tao et al. 2019 [41]
Bioaugmentation + biostimulation	Crude oil (TPH at 19.8 g/kg)	Soil microcosms	28% (biostimulation) and 14% (bioaugmentation)	12 weeks	Biostimulation allowed to achieve superior efficiency compared to bioaugmentation. Application of bioaugmentation resulted in notably decreased biodiversity of the soil community.	Wu et al. 2019 [42]
Bioaugmentation + biostimulation + surfactant-assisted biodegradation	Diesel oil hydrocarbons (3 g/kg) + PAHs (400 µg/kg)	Weathered oily-soil biopiles	39% for diesel oil hydrocarbons and 32% for PAHs	160 days	Combined bioaugmentation, biostimulation and surfactant supplementation (Tween 80) improved the biodegradation efficiency. In case of biostimulation, ammonium nitrate facilitated the process, whereas the use of urea inhibited the biodegradation efficiency.	Oualha et al. 2019 [43]
Biostimulation + surfactant-assisted biodegradation	Crude oil (either 20 g/kg or 50 g/kg)	Field study in soil	49–62%	486 days	Biostimulation improved the biodegradation efficiency in all experimental variants. Surfactant supplementation (Bioversal) improved the biodegradation process in cases of higher concentration of crude oil, whereas in cases of lower concentrations, it did not significantly affect the process.	Ortega et al. 2018 [44]
Biosurfactant-assisted biodegradation	Phenanthrene (0.1–1.0 mg/L)	Sorption reactors with soil	>90%	Up to 50 days	Supplementation of the biosurfactant (rhamnolipids) influenced the sorption kinetics of phenanthrene; however, it had no effect on its biodegradation kinetics. No significant influence of the biosurfactant on the main phenanthrene degraders was observed.	Crampon et al. 2017 [45]
Biosurfactant-assisted biodegradation	Hexadecane (2% *v*/*v*)	Flask studies	20–100%	180 h	The biosurfactant (rhamnolipids) increased the availability of hexadecane in the case of *Pseudomonas aeruginosa* (which was capable of producing rhamnolipids) and decreased the availability in the case of *Pseudomonas putida* (which was unable to produce rhamnolipids). The decrease occurred due to a blocking effect by rhamnolipids. Dissipation of rhamnolipids was also observed.	Liu et al. 2017 [46]
Biosurfactant-assisted biodegradation	Crude oil (1% *v*/*v*)	Flask studies	>85%	14 days	Isolates from beach sediments exhibited the ability to efficiently degrade hydrocarbons and produce biosurfactants. The biosurfactants increased the emulsification of crude oil and facilitated the biodegradation process.	Lee et al. 2018 [47]
Biosurfactant-assisted biodegradation	Phenanthrene (0.2–1.0 mg/L)	Flask studies	60–100%	14 days	The biosurfactant (rhamnolipids) was supplemented in order to improve the biodegradation efficiency. At a concentration of up to 100 mg/L of rhamnolipids, an enhancement of phenanthrene biodegradation was observed. At concentrations higher than 200 mg/L of rhamnolipids, the biodegradation efficiency was decreased due to the hindered biosorption of phenanthrene.	Ma et al. 2018 [48]
Biosurfactant-assisted biodegradation	PAHs: phenanthrene, fluoranthene, and pyrene (6 mg/kg)	Soil microcosms	72% for phenanthrene, 64% for fluoranthene, and 58% for pyrene at day 7	up to 35 days	Supplementation with the biosurfactant (rhamnolipids) initially increased the biodegradation of the studied PAHs (at day 7); however, no effect or even lower efficiency were observed in the latter stages (up to 35 days).	Lu et al. 2019 [49]
Biosurfactant/surfactant-assisted biodegradation	Fluorene (280 or 320 mg/L)	Flask studies	75–97%	24 h	Supplementation with the biosurfactant (rhamnolipids) allowed to achieve higher biodegradation efficiency compared to synthetic surfactants (Tween-80, Tween-60, Tween-40, Tween-20 and Triton X-100).	Reddy et al. 2018 [50]
Biosurfactant/surfactant-assisted biodegradation	Diesel oil (1% *v*/*v*)	Flaks studies	20–99%	7 days	Surfactant supplementation (Tween-80) enhanced the biodegradation of diesel oil hydrocarbons. Supplementation with the biosurfactant (rhamnolipids) inhibited the biodegradation process due to their utilization as a preferential substrate.	Staninska-Pięta et al. 2019 [51]
Natural attenuation + autochthonous bioaugmentation	Diesel oil (1% *v*/*v*)	Flask tests	20–40%	7 days	Efficiency of biodegradation processes with autochthonous bioaugmentation depended on the previous exposure of soils to pollution. In the majority of tested systems, the autochthonous bioaugmentation resulted in a significant enrichment of *Proteobacteria*.	Czarny et al. 2019 [52]
Natural attenuation + bioaugmentation + biostimulation	Engine oil (39–41 g/kg TPH)	Soil microcosms	31–75%	210 days	The combined bioaugmentation and biostimulation approach resulted in the inhibition of biodegradation processes in comparison to natural attenuation.	Ramadass et al. 2018 [53]
Natural attenuation + bioaugmentation + biostimulation	Petroleum refinery waste (TPH at 144 g/kg)	Vial microcosms	57–75%	120 days	Combined bioaugmentation-biostimulation approach allowed to achieve the best biodegradation efficiency. Biostimulation was the major driving force for the enhancement.	Roy et al. 2018 [54]
Natural attenuation + bioaugmentation + biostimulation	Crude oil (20 g/kg)	Bioreactors with soil	51–90%	60 days	The combined bioaugmentation and biostimulation approach allowed to achieve the highest biodegradation rate. Among individual treatments, the efficiency of biostimulation was superior (82% of TPH removal) compared to bioaugmentation (63% of TPH removal).	Safdari et al. 2018 [55]
Natural attenuation + bioaugmentation + biostimulation	Crude oil (3% *w*/*v*)	Soil microcosms	94%	45 days	Combined bioaugmentation and biostimulation allowed to achieve the most rapid and efficient biodegradation process.	Varjani and Upasani 2019 [56]
Surfactant-assisted biodegradation	PAHs (574 mg/kg)	Soil microcosms	72–77%	84 days	Enhanced biodegradation was observed at sub-CMC concentrations of the surfactant (Triton X-100), whereas decreased efficiency was observed at CMC concentrations. The negative effect may be attributed to the preferential degradation of surfactant at CMC concentrations.	Cecotti et al. 2018 [57]
